# A dyadic perspective on trust in physicians and quality of life in pediatric asthma: an actor–partner interdependence model on children and their parents

**DOI:** 10.1093/jpepsy/jsag017

**Published:** 2026-03-14

**Authors:** Serena Petrocchi

**Affiliations:** Institute of Family Medicine, Università della Svizzera italiana, Lugano, Switzerland

**Keywords:** trust, quality of life, asthma, children, parents, dyadic data, APIM Data is available on request

## Abstract

**Objectives:**

This study examined the effects of dyadic trust in physicians as a determinant of children’s quality of life (QoL) in pediatric asthma. Actor and partner effects of children’s and parents’ trust towards physician were tested, and linear vs. curvilinear associations were compared within an Actor–Partner Interdependence Model (APIM).

**Methods:**

A longitudinal design included 222 child–mother dyads (children’s *M*_age_ = 12.6 years) assessed at two time points 1 year apart. Children completed trust in physician scale and a QoL questionnaire; parents reported their own trust in physicians, asthma severity, adherence, and illness-related conflict with the child.

**Results:**

The linear APIM demonstrated a good fit (χ^2^(12) = 24.53, Comparative Fit Index (CFI) = 0.97, Tucker-Lewis Index (TLI) = 0.93, Root Mean Square Error of Approximation (RMSEA) = 0.07, Standardized Root Mean Square Residual (SRMR) = 0.08) and explained 50% of the variance in children’s QoL at Time 2. Both actor (β = .18, *p* = .018) and partner (β = .18, *p* = .007) effects of trust were significant and statistically equivalent (Wald χ^2^ (1) = 0.25, *p* = .62). The quadratic model showed poorer fit (CFI = 0.89, RMSEA = 0.11), and model comparisons favored the linear specification (Δχ^2^ (2) = 8.63, *p* = .013).

**Discussion:**

Findings highlight children’s and mothers’ trust in physicians as a dyadic, mutually reinforcing predictor of children’s well-being. In pediatric asthma, higher trust, whether from the child or the parent, consistently benefits QoL, supporting a linear rather than curvilinear relationship. Strengthening trust on both sides of the parent–child dyads may enhance adherence, engagement, and long-term health outcomes.

Pediatric asthma, a prevalent chronic childhood disease, is associated with challenges in physical, psychological, and social functioning (Silva et al., 2015). Despite declines in mortality, asthma continues to pose a meaningful burden for many children, highlighting the importance of effective management strategies (Asher et al., 2021). The quality of life (QoL) in children with asthma is a multifaceted construct influenced by various clinical, psychological, and sociocultural factors. A systematic review by Everhart and Fiese (2009) found a significant relationship between asthma severity and child QoL. Other studies show that uncontrolled asthma is often associated with poorer QoL (Ali, 2023; Banjari et al., 2018). Beyond physiological impacts, family relational difficulties have been linked to challenges in disease management and may be associated with poorer QoL (Plaza-González et al., 2022). Petsios et al. (2011) examined the agreement levels between parents and asthmatic children regarding health-related QoL, providing insight into the dyadic perception of QoL. Social determinants of health, such as socioeconomic status and access to care, have been shown to play an important role in children’s health outcomes and QoL (Federico et al., 2020; Horner, 2020). Finally, adequate medication adherence is central to achieving symptom control and preserving QoL (Boutopoulou et al., 2018; McCrossan et al., 2024; Searle et al., 2017).

Asthma management in childhood typically involves a collaborative parent–child process, with parents often taking primary responsibility for monitoring triggers, symptoms, and medication use. However, these routines vary considerably across families and evolve as children develop, with children gradually assuming greater involvement in self-management as their skills and autonomy increase (Miller, 2009; Sleath et al., 2011). These considerations point to the relevance of parent–child dyadic dynamics and psychosocial factors in understanding adherence behaviors and children’s QoL.

Among the psychosocial factors, trust in the physician emerges as a critical element. Trust is defined as the patient’s willingness to be vulnerable based on the belief that the doctor will perform benevolent actions toward the patient (Moseley et al., 2006). Illness often involves experiences of vulnerability, which can increase the relevance of supportive and coordinated medical care. Research has extensively investigated trust in adult patients. Greater trust in a physician is linked to better treatment adherence, greater willingness to follow medical advice, and fewer conflicts between doctor and patient (Hall et al., 2002; Thom et al., 2011). According to a meta-analysis (Birkhäuer et al., 2017), trust has a positive and moderate correlation with subjective health outcomes and patient satisfaction. It also promotes patients’ and doctors’ openness and collaboration in clinical encounters (Rinaldi et al., 2025b, 2025c), as well as medication adherence (Reid et al., 2008).

Research on children’s trust in physicians, while growing, remains limited compared to the extensive literature on adult populations. First, trust has been measured in pediatric care with a scale demonstrating good psychometric properties and validity (Rotenberg et al., 2008). Second, in pediatric asthma, it has been found that children’s trust in physicians positively influences QoL and adherence (Rotenberg & Petrocchi, 2018). Third, parents’ trust in the physician influenced children’s QoL and adherence (Rotenberg & Petrocchi, 2018). Fourth, both linear and quadratic relationships have been identified between children’s and parents’ trust in physicians and children’s QoL (Petrocchi & Rotenberg, 2024). Notwithstanding, there are gaps in (1) considering more than one reporter of trust toward a doctor (only the child vs. only the parent) and understanding the bidirectional nature of trust within the parent–child dyad towards physicians, (2) providing longitudinal evidence (MacKay et al., 2024) that connects trust and QoL over time. This study addresses these gaps by longitudinally examining the dyadic interplay of parent and child trust in physicians within the context of pediatric asthma, applying an Actor–Partner Interdependence Model to assess their reciprocal influences on QoL over time (the so-called actor and partner effects). The following research questions/hypotheses have been formulated (see Figure 1):

**Figure 1.**
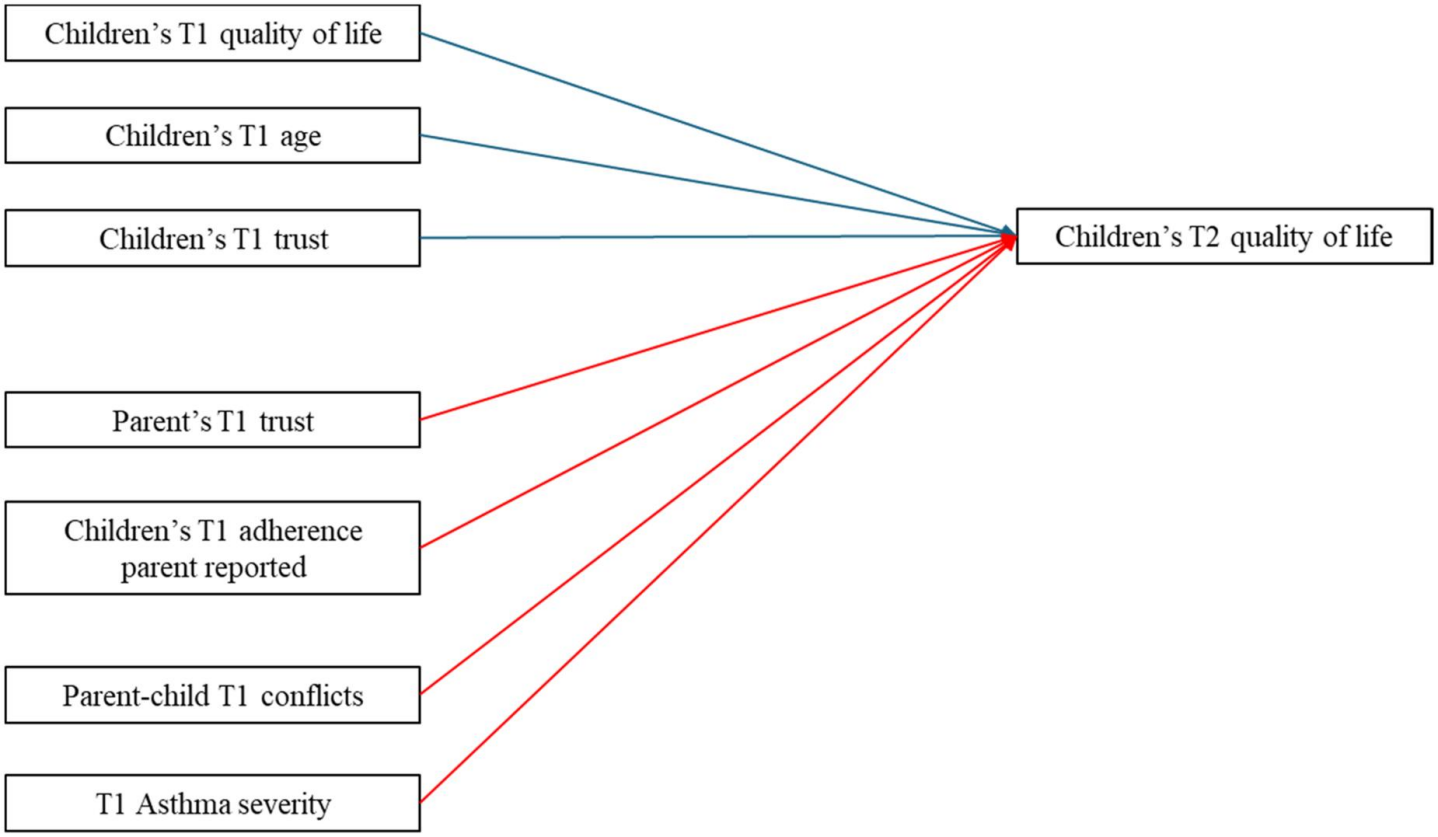
Theoretical model tested.

HP1. It was hypothesized that children’s trust in their physician at Time 1 would positively influence the children’s QoL at Time 2 (significant actor effect), controlling for the partner effect. In the context of parent–child dyads, actor effects are expected because a child’s own trust directly influences their engagement with treatment and QoL (Rotenberg & Petrocchi, 2018).

HP2. It was hypothesized that parents’ trust at Time 1 would positively influence the child’s QoL at Time 2 (significant partner effect), controlling for the actor effect. Partner effects are also anticipated as trust is a dyadic and dynamic phenomenon, emerging through ongoing communication and relational behaviors (Elwood, 2023), including those between parents and their child’s physician. Parents often advocate for their child’s optimal care and treatment, and their trust in the physician can significantly influence their child’s engagement with the healthcare plan. Parents’ trust in the physician can enhance their child’s QoL by promoting adherence to recommended treatment strategies.

RQ1. To what extent do actor effects demonstrate a greater magnitude or relative influence compared to partner effects? This is the first time such a comparative analysis has been conducted, examining the relative influence of individual trust perceptions vs. the indirect effects of a dyadic partner’s trust on the child’s QoL.

RQ2. To what extent do linear vs. quadratic models of the relationship between trust and QoL account for greater variability in outcomes? Linear relationships are expected due to the direct relationship between increased trust and enhanced QoL in non-dyadic studies (Rotenberg & Petrocchi, 2018). Conversely, quadratic relationships suggest that the impact of trust on QoL may not be uniformly linear. In contrast, moderate levels of trust are beneficial; excessively low or very high levels might yield diminishing returns or even detrimental effects, indicating a more complex, non-constant influence (Petrocchi & Rotenberg, 2024). Linear vs. quadratic relationships between trust and QoL have never been tested in an APIM.

## Materials and Methods

### Participants

At Time 1 (T1), 222 children (178 males) and their mothers were tested. The children’s *M* age was 12 years 6 months (*SD* = 1.6 years), and the age ranged from 8 to 16 years. The same sample was tested at Time 2 (T2) approximately 1 year later. At T1, a total of 63.6% of participants provided a valid postal code; 36.0% provided an invalid one, and 0.4% (*n* = 1) had missing data. Participants were primarily from England (81%), with smaller proportions from Scotland (9.9%), Wales (5.7%), and Northern Ireland (2.8%). At T1, participants also provided information regarding the therapeutic plan they were following and the care setting. See Table 1 for descriptive information about the sample and Table 2 for basic statistics.

**Table 1.** Sample characteristics.

Variables	*M* (*SD*) or f%
Age	12.5 (1.6)
Gender	80% males
Parent-rated asthma problem 1 (T1)	4.92 (1.09)
Parent-rated asthma problem 2 (T1)	4.47 (1.02)
Parent-rated asthma problem total (T1)	4.69 (.98)
Asthma medication (T1)	
No regular asthma medication	1.8%
SABA only	6.3%
Regular ICS	39.6%
ICS + LABA	40.4%
LTRA	10.8%
Living area (T1)	
London	14.9%
Large urban cities	53.2%
Medium urban cities	26.2%
Rural/semi-rural areas	5.7%
Care setting (T1)	
General practitioner	54.5%
Specialist clinics	41.0%

*Note*. *f*% = percentage; *ICS* = inhaled corticosteroids; *LABA* = long-acting β2-agonist; *LTRA* = leukotriene receptor antagonist; *M* = mean; *SABA* = short-acting β2-agonist. Parent-rated asthma problem 1/2 = How often your child misses school due to asthma attacks? (item 1) and How often your child experiences asthma attacks in general? (item 2). Parent-rated asthma problem total is the composite parent-rated asthma problem score derived by calculating the *M* of the two items.

**Table 2.** Basic statistics.

Variables	Min	Max	*M*	*SD*
Children’s T1 trust	24.00	42.00	33.95	4.13
Children’s T2 trust	21.00	44.00	31.85	3.61
T1 Quality of life	1.33	6.08	5.04	0.80
T2 Quality of life	1.08	6.50	4.94	0.82
T1 Adherence	2.00	4.38	3.53	0.55
Parents’ T1 trust	8.00	25.00	19.05	3.18
Parents’ T2 trust	9.00	25.00	16.96	2.47

### Measures

#### Trust in physicians

For pediatric patients, trust was evaluated using the Children’s Trust in General Physicians Scale (Rotenberg et al., 2008). It consists of nine hypothetical scenarios describing everyday interactions between a child and a general figure of a doctor. This scale measures children’s trust across three bases—reliability, emotional trust, and honesty—and calculates it as one general dimension (Rotenberg, 2025). Reliability measures whether children think that a physician fulfills their promises or responds promptly, emotional trust measures how much children believe their physician keeps their confidence private, and honesty measures whether the child believes their physician is truthful (Rotenberg & Petrocchi, 2018). Response options ranged from 1 “*strongly disagree*” to 5 “*strongly agree*”, with higher scores indicating greater levels of trust in physicians. The scale has demonstrated strong psychometric properties in previous research with pediatric populations, exhibiting robust internal consistency and construct validity (Rotenberg & Petrocchi, 2018); internal consistency was α = .60 at T1 and α = .69 at T2 and moderate test–retest stability across 1 year, *r*(141) = 0.36, *p* < .001. Maternal trust in physicians was assessed using the Trust in Physicians Scale, which is a widely adopted measure in adult populations (Hall et al., 2002). Two items referring to payment for health services were dropped since their limited relevance in the U.K. healthcare system. The scale includes nine statements about a general doctor’s behavior, attitudes, and professionalism (e.g., honesty, competence, attentiveness, confidentiality). Response options ranged from 1 “*strongly disagree*” to 5 “*strongly agree*,” with higher scores indicating greater trust. Previous research has demonstrated acceptable psychometric properties (Rotenberg & Petrocchi, 2018) (α = .64 at T1 and α = .68 at T2 and small but significant test–retest stability across 1 year, *r*(141) = 0.22, *p* < .001).

#### Quality of life

The 23‐item Pediatric Asthma Quality of Life Questionnaire (PAQLQ; Juniper et al., 1996) assesses the problems that asthmatic children experience in their daily lives. The PAQLQ has been found to demonstrate acceptable internal consistency (overall Cronbach’s αs > 0.70) and stability, as well as construct validity by its association with conventional asthma indices and general QoL (Hafkamp-de Groen & Raat, 2012; Juniper et al., 1996). The PAQLQ in the current study demonstrated acceptable internal consistency (αs = 0.77 and 0.83 at T1 and T2, respectively) and stability across 1 year, *r*(141) = 0.72, *p* < .001.

#### Covariates

To control the source of bias, at T1, parents reported their child’s adherence, the impact of asthma symptoms on daily functioning, and parent–child illness-related conflict. They rated the extent to which their child adhere to asthma care across seven behaviors (adapted from Hayford & Ross, 1988): uses inhalers as required, recalls doctor’s appointments, attempts to control breathing to prevent an attack, avoids things he or she is allergic to, takes medications as prescribed by the physician, adheres to the physician-prescribed diet, and adheres to the physician-prescribed exercise regime. Each behavior was rated on a 5-point scale (1 = *Completely*, 2 = *Usually*, 3 = *Somewhat*, 4 = *Only a bit*, 5 = *Not at all*) with a *Not Applicable* (8) option. The 7 items were averaged so that higher scores represented greater adherence to prescribed medical regimes (with acceptable internal consistency given the modest number of items, α = 0.50). Furthermore, two items asked parents to assess the impact of asthma symptoms on the child’s daily functioning (How often your child misses school due to asthma attacks? and How often your child experiences asthma attacks in general?). Responses are rated on a 7-point Likert scale, ranging from 1 = *Every day* to 7 = *Hardly ever*, indicating the severity and control of the child’s asthma over time. The total score was reversed, with higher scores indicating more frequent asthma attacks and school absences, reflecting worse asthma control. One item assessed parent–child illness-related conflict as reported by parents. The item measures the frequency of disagreements or tensions between parent and child about the child’s illness (rate the frequency with which you and your child experience conflict regarding his or her illness). Response options ranged from 1 = *very frequently* to 5 = *hardly ever*. The score was reversed.

### Procedure and contextual information

Participants were recruited through two organizations: the University Hospitals of North Midlands and Asthma + Lung UK. The University Hospitals of North Midlands is a National Health Service (NHS) provider offering medical care to children with asthma in the North Midlands region of the UK. Families attending clinical visits were approached by trained research and medical staff, who explained the purpose of the study and invited them to participate. Asthma + Lung UK is a leading UK charity providing support and consultancy services for individuals with asthma and their caregivers; families affiliated with the organization were contacted via private email invitations.

Parents and children were provided with detailed information sheets describing the study. Parents gave written informed consent for their own participation and for their child’s participation, and children provided assent. At Time 1 (T1), parents and children completed the questionnaires via a secure online link. Approximately 1 year later, they were invited to complete Time 2 (T2) questionnaires using the same procedure. Ethical approval was obtained from the University Ethical Review Panel and the National Research Ethics Service UK (10/H1017/53). Families received a £10 gift card as compensation for their time following completion of T2. Portions of the dataset have been reported previously (Rotenberg & Petrocchi, 2018, 2024); however, the present analyses focus on the full available sample.

Children were eligible if they had a physician-diagnosed asthma condition, were between 8 and 16 years old, could provide assent, and could complete the questionnaires with parental support as needed. Parents were eligible if they were the child’s primary caregiver and able to provide informed consent. No additional clinical or demographic exclusion criteria were applied, allowing for the recruitment of a naturalistic sample of families managing pediatric asthma.

In the United Kingdom, pediatric asthma care is embedded within the NHS, where general practitioners (GPs) act as gatekeepers and coordinators of care. Children with asthma are typically offered at least one structured annual review in primary care, with more frequent planned follow-ups (every 3–6 months) when symptoms are not well controlled or when treatment adjustments are required. Specialist asthma services, accessed through GP referral, provide closer monitoring for children with more complex or higher-risk profiles, often scheduling follow-up visits approximately every 3 months.

### Data analysis strategy

At T1, missing data were minimal for most variables: age had 10% missing values, asthma medication had 1%, and care setting had 4.5%. The area of residence had a missing rate of 36.4%, due to invalid or unreported postcodes. The psychological measures and the evaluation of asthma severity had less than 1% of missing data. Similarly, at T2, missing data for the variables included in the model were below 5%.

An Actor–Partner Interdependence Model was estimated in RStudio v.2025.09.1 + 401 with the Lavaan package (Rosseel, 2012; Rosseel et al., 2025; see Supplementary material for a detailed explanation of the tested model) using the Maximum Likelihood Robust estimator, which provides robust *SEs* and fit indices that are adjusted for potential non-normality in the observed variables and Full-Information Maximum Likelihood (FIML) for missing data. FIML estimation allows for the inclusion of cases with partially missing data under the assumption that data are missing at random.

Dyads were distinguishable by role and analyzed in a wide data format. The structural model included actor and partner effects of trust from T1 to T2 for both child and parent on child’s QoL at T2, controlling for baseline QoL, child’s age, gender, and covariates. The covariances among baseline predictors and residual covariances were allowed to vary. Model fit was assessed with chi-square, CFI/TLI, RMSEA with 90% confidence interval (CI), and SRMR; standardized coefficients and *R*^2^ were reported for interpretability. To test whether actor effects exceeded partner effects, we conducted Wald tests on equality constraints for T1-to-outcome. Modification indices were inspected but not used to respecify the model.

## Results

**Figure 2.**
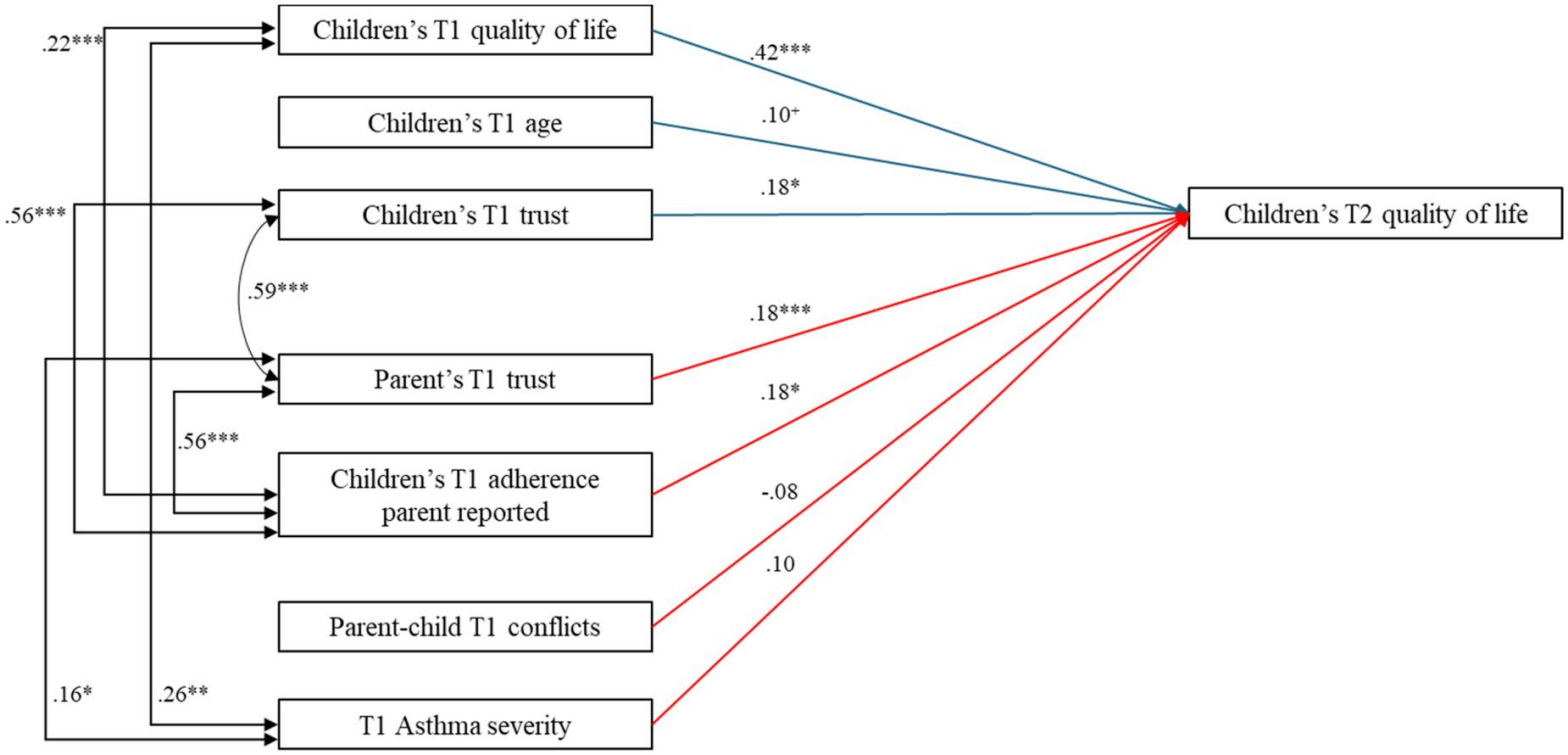
Results of the tested model. Notes: the other covariances did not reach statistical significance. + *p* = .07, * *p* < .05, ** *p* < .01, *** *p* < .001. Blue lines indicate actor effects. Red lines indicate partner effects.

The linear APIM model fit was acceptable, χ^2^(12) = 24.53, *p* = .017, CFI = 0.97, TLI = 0.93, RMSEA = 0.07 (90% CI 0.03–0.11), and SRMR = 0.08. The scaled robust fit indices showed a similar pattern, with χ^2^(12) = 22.98, *p* = .028, robust CFI = 0.96, robust TLI = 0.92, and robust RMSEA = 0.08 (90% CI 0.04–0.12). The model accounted for approximately 50% of the variance in the child’s QoL at Time 2 (*R*^2^ = 0.50). See Figure 2 and Table 1.

The child’s QoL at Time 2 was significantly predicted by the child’s trust in the doctor (actor effect, β = .18, *b* = 0.034, *SE* = 0.014, 95% CI 0.006–0.062, *p* = .018), the parent’s trust in the doctor (partner effect, β = .18, *b* = 0.045, *SE* = 0.017, 95% CI 0.012–0.078, *p* = .007), the child’s T1 QoL (actor autoregressive effect, β = .42, *b* = 0.416, *SE* = 0.108, 95% CI 0.204–0.628, *p* < .001), and T1 adherence parent-reported (β = .18, *b* = 0.259, *SE* = 0.105, 95% CI 0.053–0.465, *p* = .014). The effect of the child’s age approached significance (β = .10, *b* = 0.004, *SE* = 0.002, 95% CI –0.0001 to 0.008, *p* = .057). The parent’s reported asthma problems (β = .10, *b* = 0.081, *SE* = 0.063, 95% CI –0.043 to 0.205, *p* = .195) and illness-related conflict (β = –.08, *b* = –0.078, *SE* = 0.043, 95% CI –0.162 to 0.006, *p* = .073) were not significant predictors.

A Wald test comparing the actor and partner effects indicated that the two coefficients did not differ significantly, χ^2^(1) = 0.25, *p* = .62. This suggests that the strength of the child’s trust and the parent’s trust in predicting the child’s later QoL was statistically equivalent.

The quadratic APIM showed poor fit to the data, χ^2^(19) = 71.79, *p* < .001, CFI = 0.89, TLI = 0.74, RMSEA = 0.11, 90% CI 0.09–0.14, SRMR = 0.08. Robust fit indices were similar (robust CFI = 0.88, robust TLI = 0.71, robust RMSEA = 0.12, 90% CI 0.09–0.15). The model explained 48% of the variance in child QoL at Time 2 (*R*^2^ = 0.48). The quadratic effect of child trust was significant (*b* = –0.006, *SE* = 0.002, β = –.14, *p* = .014), whereas the quadratic effect of parent trust was not (*b* = –0.006, *SE* = 0.005, β = –.08, *p* = .240).

A nested model including both linear and quadratic effects for parent and child trust provided a slightly improved fit, χ^2^(17) = 52.76, *p* < .001, CFI = 0.93, TLI = 0.81, RMSEA = 0.10, 90% CI .07–0.13, SRMR = 0.08. The robust indices again indicated similar fit (robust CFI = 0.91, robust TLI = 0.77, robust RMSEA = 0.10, 90% CI .07–.14). The model accounted for 54% of the variance in child QoL at Time 2 (*R*^2^ = 0.54). In this model, the linear effects of both children (*b* = 0.032, *SE* = 0.014, β = .17, *p* = .017) and parent trust (*b* = 0.041, *SE* = 0.018, β = .16, *p* = .024) were significant. In contrast, the quadratic term for children’s trust approached significance (*b* = –0.005, *SE* = 0.002, β = –.11, *p* = .054).

A scaled chi-square difference test comparing the purely quadratic model with the full (nested) model that included both linear and quadratic effects indicated a significant improvement in fit when linear effects were added, Δχ^2^(2) = 8.63, *p* = .013. This suggests that linear trust effects contribute meaningfully beyond the quadratic components. Wald tests further supported this interpretation. The joint test of the quadratic terms was marginally significant, Wald χ^2^(2) = 5.20, *p* = .074, whereas the joint test of the linear effects was significant, Wald χ^2^(2) = 13.08, *p* = .001. These results indicate that the dyadic linear components of trust are stronger and more reliable predictors of child QoL than the dyadic quadratic terms, supporting a primarily linear relationship between trust and QoL over time.

## Discussion

Consistent with hypothesis 1, the child’s trust in the physician at Time 1 significantly predicted the child’s QoL at Time 2, even after controlling for baseline functioning and parent-related variables. This significant actor effect indicates that greater child trust in the physician is associated with better subsequent well-being. This result aligns with and expands Rotenberg and Petrocchi (2018), who argue that children’s interpersonal trust facilitates emotional security and engagement in care, thereby enhancing adherence and adjustment (Street et al., 2009). Within the healthcare context, children who perceive their physician as reliable, honest, and emotionally supportive may experience reduced anxiety about treatment and show more positive health-related behaviors as seen in adults (Birkhäuer et al., 2017), contributing to improved QoL over time.

Similarly, results also supported hypothesis 2: the parents’ trust in the physician at Time 1 had a significant partner effect on the child’s later QoL. This finding underscores the dyadic and relational nature of trust within families managing chronic illness. As Elwood (2023) highlights, trust emerges through communicative and relational processes that shape cooperation and health engagement (Epstein & Street, 2011). Parents with higher trust in the physician are more likely to follow medical recommendations (Hall et al., 2002), communicate effectively with healthcare providers (Thom et al., 2004, 2011), and foster a supportive home environment for treatment adherence (Petrocchi & Rotenberg, 2024), all mechanisms that can indirectly enhance the child’s physical and emotional well-being.

Regarding research question 1, comparing the magnitudes of the actor and partner effects revealed no statistically significant difference between them. This indicates that the child’s own trust and the parent’s trust contribute equally to the child’s later QoL. This finding is theoretically important: it demonstrates that trust is not only an individual psychological resource but also a dyadic one (Petrocchi et al., 2019). The equivalence of actor and partner effects suggests that both members of the dyad actively co-construct a trusting context around medical care, and both forms of trust, personal and indirect, hold comparable weight in influencing the child’s health outcomes.

The analyses addressing research question 2 further clarify the nature of this relationship. Although previous studies have explored the association between trust and QoL at an individual level, none have examined the linear vs quadratic nature of these relationships in dyadic contexts. Rotenberg and Petrocchi (2018) found support for a linear relationship, where higher levels of interpersonal trust are consistently linked to better QoL. In contrast, a more recent empirical work by Petrocchi and Rotenberg (2024) suggests that, in some circumstances, curvilinear associations may emerge, where very low or excessively high trust could have diminished effects. The current study extends this line of research by testing both linear and quadratic effects of trust within a dyadic APIM. Results clearly favored a linear model, with both actor and partner effects of trust significantly predicting the child’s later QoL, while quadratic terms added no meaningful explanatory value. This indicates that, within the parent–child–physician triad, higher levels of trust, whether from the child or the parent, are consistently beneficial. Because the present study employed a dyadic model, it captures shared and reciprocal processes, such as mutual validation and modeling of trust between parent and child (Houtum et al., 2021; Rotenberg, 2025), that may stabilize the effects of trust and prevent the extremes seen in individual-level data. Future studies should continue to test these conditions by examining whether non-linear trust dynamics emerge in dyads characterized by lower communication quality, greater conflict, or divergent illness perceptions. These results suggest that efforts to cultivate trust should not target only the patient but also extend to the parent–physician relationship. Interventions that emphasize transparency, empathy, and shared decision-making can strengthen trust on both sides of the dyad, thereby promoting adherence and improving children’s overall QoL. In this line, future research should extend these findings by testing longitudinal cross-lagged APIMs, exploring potential mediators such as adherence and communication quality, and examining moderators such as illness severity or child/family-healthcare provider conflicts (see Elwood, 2023).

Several limitations should be acknowledged. First, the sample consisted of mother–child dyads, limiting the generalizability of the findings to other caregivers such as fathers who may play different roles in asthma management. Second, the measures relied on self-report questionnaires, which may introduce social desirability and shared method variance. Although self-report measures introduce potential biases, constructs such as trust and QoL are inherently subjective and cannot be reliably assessed through alternative methods. Nevertheless, future studies would benefit from incorporating multi-informant assessments for variables for which external validation is feasible, such as treatment adherence (e.g., clinician ratings, parent reports, and child reports), as well as objective markers of asthma severity (e.g., PEAK flow or spirometry data). Finally, trust was measured using a general physician trust scale, rather than a provider-specific assessment; therefore, a specialty-specific trust measure could offer greater precision and provide a direction for future research. Third, although longitudinal design improves causal inference, the study included only two time points, preventing the examination of more complex reciprocal dynamics over time. Future research should consider the inclusion of multiple assessment waves to allow for cross-lagged or longitudinal mediation analyses that can better capture bidirectional and dynamic processes. In addition, the internal consistency of some measures (e.g., adherence and parental trust) was modest, which may have attenuated effect sizes. Fourth, the study did not include socioeconomic or neighborhood-level indicators, which limits the ability to account for broader contextual influences. However, the QoL measure used here captures asthma-specific functioning, which is more directly related to disease management and may be less sensitive to wider socioeconomic variation.

Finally, as the study was conducted within the UK healthcare system, the findings may not be fully generalizable to countries with different healthcare structures. The UK’s universal system, with GPs acting as gatekeepers and providing high continuity of care, may shape relational processes such as parent–child trust in physicians, potentially influencing the patterns observed in this study. However, research conducted in other countries (see also Betts et al., 2014), Italy (a publicly funded national health system; Petrocchi et al., 2019), Switzerland (an insurance-based model; Rinaldi et al., 2025a, 2025b, 2025c), and the United States (a mixed system; Hall et al., 2001; Mechanic & Meyer, 2000; Thom, 1999), consistently identifies trust as a key determinant of engagement, adherence, and QoL. This convergence across structurally different health systems supports the broader relevance of our findings while acknowledging that system-level factors may influence the strength or expression of these dyadic processes.

## Conclusion

The present study highlights the central role of trust in the physician as a dyadic determinant of children’s QoL in chronic illness management. Both the child’s and the parent’s trust exerted significant and comparable effects on later well-being, supporting the idea that trust operates not only as an individual attitude but as a shared interpersonal process within families. This is the first study to compare linear vs. quadratic trust–QoL associations in a dyadic framework, providing a novel empirical contribution to the trust literature and confirming that in pediatric health contexts, trust exerts a robust, linear, and mutually reinforcing effect on well-being. This reinforces prior conceptualizations of trust as a fundamental and continuously beneficial resource in pediatric care. The findings suggest that interventions designed to enhance trust, through improved communication, transparency, and shared decision-making, may have meaningful effects on children’s QoL and likely adherence and engagement. These findings underscore the need for pediatric care models that actively engage both children and parents in building and maintaining trusting, collaborative relationships with healthcare providers.

Several evidence-based strategies have been shown to foster trust in medical settings. Physician behaviors such as empathic and patient-centered communication, active listening, transparency about treatment options, and shared decision-making reliably increase patients’ trust (Epstein & Street, 2011; Street et al., 2009; Thom et al., 2004). Continuity of care also plays a key role, with patients reporting higher trust when they consistently see the same clinician (Mainous et al., 2001; Saultz & Albedaiwi, 2004). Training programs focused on enhancing clinicians’ empathic skills have similarly been shown to improve perceived empathy and patient trust (Riess et al., 2012). These strategies are relevant not only for child–physician interactions but also for parent–physician relationships, both of which shape the child’s health outcomes.

## Supplementary Material

jsag017_Supplementary_Data

## Data Availability

The data underlying this article will be shared on reasonable request to the corresponding author.
