# Supplementary material for: A dyadic perspective on trust in physicians and quality of life in pediatric asthma: an actor–partner interdependence model on children and their parents
Source: J Pediatr Psychol. 2026 Mar 14;51(7):593–600. doi: 10.1093/jpepsy/jsag017 (PMC13373574; doi:10.1093/jpepsy/jsag017)
Supplement: jsag017_Supplementary_Data [file jsag017_supplementary_data.zip › jpepsy-2025-0240-File007.docx]

**Supplementary material**

- **Results of the Confirmatory Factor Analysis**

Using the sample included in this paper, a confirmatory factor structure was tested for the Children’s Trust in General Physicians Scale (CTGP; Rotenberg et al., 2008) and one for the Trust in Physicians Scale (Hall et al., 2002) administered to the mothers.

The CTGP showed the expected one-factor structure with a good fit of the data, χ2(17) = 19.66, p = .29, CFI = .97, TLI = .95, RMSEA = .029, 90% CI [.000, .072], SRMR = .044. Similarly, the TPS showed the one-factor structure as expected, χ2(24) = 34.55, p = .07, CFI = .94, TLI = .91, RMSEA = .05, 90% CI [.000, .08], SRMR =.05.

- **RStudio script**

**1)Data preparation**

## 0) Packages

library(lavaan)

## 1) Load the long-format dataset

## 2) Normalize the dyad key and check the role variable

stopifnot(all(c("dyad","role") %in% names(trust_long)))

trust_long$diade <- trimws(as.character(trust_long$diade))

table(trust_long$role, useNA = "ifany") # expected: 1 = parent, 2 = child

## 3) Split the long data by role

parents <- subset(trust_long, role == 1)

children <- subset(trust_long, role == 2)

## 4) Merge into wide format

apim_data <- merge(parents, children, by = "dyad",

suffixes = c("_par","_ch"),

all = FALSE)

cat("Rows after merge:", nrow(apim_data), "\n")

## 5) Check that all variables required for the model are present

expected <- c(

# predictors / control variables

"trust1_par","trust1_ch",

"Adh1_val_par","QoLT1_ch","age_ch",

"asthmaproblem_par","conflicts_par",

# outcomes

"QoLT2_val_ch")

missing_vars <- setdiff(expected, names(apim_data))

if (length(missing_vars) > 0) {

stop("The following variables are missing after the merge: ",

paste(missing_vars, collapse = ", ")) }

**2)Model Script for the linear relationships**

## 6) lavaan model

model <- '

QoLT2_val_ch ~ a1*trust1_ch + c1*QoLT1_ch + age_ch + asthmaproblem_par + conflicts_par

QoLT2_val_ch ~ p1*trust1_par + d2*Adh1_val_par + asthmaproblem_par + conflicts_par

trust1_par ~~ trust1_ch

Adh1_val_par ~~ QoLT1_ch

trust1_par ~~ Adh1_val_par

Adh1_val_par ~~ trust1_ch

QoLT1_ch ~~ trust1_ch

age_ch ~~ Adh1_val_par

age_ch ~~ QoLT1_ch

QoLT1_ch ~~ asthmaproblem_par

trust1_par ~~ asthmaproblem_par

'

## 7) Model estimation

fit_apim <- sem(model,

data = apim_data,

missing = "fiml",

estimator = "MLR")

## Model summary

summary(fit_apim, fit.measures = TRUE, standardized = TRUE, rsquare = TRUE)

**3)Comparison between actor and partner effects**

## 8) Actor (a1) vs Partner (p1) comparison

# Equality test (unstandardized): H0: a1 = p1

lavTestWald(fit_apim, constraints = "a1 - p1 == 0")

# Unstandardized and standardized estimates

pe <- parameterEstimates(fit_apim, standardized = TRUE)

ap <- subset(

pe,

lhs == "QoLT2_val_ch" & op == "~" & label %in% c("a1","p1"),

select = c(label, est, se, pvalue, std.all)

)

print(ap) # est = unstandardized coefficient; std.all = standardized coefficient

# Difference (actor - partner)

diff_unstd <- with(ap, est[label == "a1"] - est[label == "p1"])

diff_std <- with(ap, std.all[label == "a1"] - std.all[label == "p1"])

cat("Diff (actor - partner), unstandardized:", diff_unstd, "\n")

cat("Diff (actor - partner), standardized: ", diff_std, "\n")

lavTestWald(

fit_apim,

constraints = "a1 - p1 == 0"

)

**4)Model Script for the quadratic relationships**

## 9) Center the predictor variables

apim_data$trust1_ch_c <- as.numeric(scale(apim_data$trust1_ch, center = TRUE, scale = FALSE))

apim_data$trust1_par_c <- as.numeric(scale(apim_data$trust1_par, center = TRUE, scale = FALSE))

## 10) Create orthogonalized quadratic terms (residual centering)

apim_data$trust1_ch_c2_orth <- resid(lm(I(trust1_ch_c^2) ~ trust1_ch_c, data = apim_data))

apim_data$trust1_par_c2_orth <- resid(lm(I(trust1_par_c^2) ~ trust1_par_c, data = apim_data))

## 11) lavaan model

model_quad <- '

QoLT2_val_ch ~ qa1*trust1_ch_c2_orth +

qp1*trust1_par_c2_orth +

c1*QoLT1_ch + age_ch + asthmaproblem_par + conflicts_par + d2*Adh1_val_par

trust1_par_c ~~ trust1_ch_c

trust1_par_c2_orth ~~ trust1_ch_c2_orth

trust1_par_c ~~ trust1_ch_c2_orth + trust1_par_c2_orth

trust1_ch_c ~~ trust1_ch_c2_orth + trust1_par_c2_orth

Adh1_val_par ~~ QoLT1_ch + trust1_ch_c + trust1_par_c + trust1_ch_c2_orth + trust1_par_c2_orth

QoLT1_ch ~~ trust1_ch_c + trust1_par_c + trust1_ch_c2_orth + trust1_par_c2_orth

age_ch ~~ Adh1_val_par + QoLT1_ch

QoLT1_ch ~~ asthmaproblem_par

trust1_par_c ~~ asthmaproblem_par

'

## 12) Model estimation

fit_apim2 <- sem(

model = model_quad,

data = apim_data,

missing = "fiml",

estimator = "MLR"

)

## 13) Output

summary(fit_apim2, fit.measures = TRUE, standardized = TRUE, rsquare = TRUE)

**5)Model Script for the nested model with both linear and quadratic relationships**

## 14) Full model (linear + quadratic effects)

model_both <- '

QoLT2_val_ch ~ a1*trust1_ch_c + p1*trust1_par_c +

qa1*trust1_ch_c2_orth + qp1*trust1_par_c2_orth +

c1*QoLT1_ch + age_ch + asthmaproblem_par + conflicts_par + d2*Adh1_val_par

trust1_par_c ~~ trust1_ch_c

trust1_par_c2_orth ~~ trust1_ch_c2_orth

trust1_par_c ~~ trust1_ch_c2_orth + trust1_par_c2_orth

trust1_ch_c ~~ trust1_ch_c2_orth + trust1_par_c2_orth

Adh1_val_par ~~ QoLT1_ch + trust1_ch_c + trust1_par_c + trust1_ch_c2_orth + trust1_par_c2_orth

QoLT1_ch ~~ trust1_ch_c + trust1_par_c + trust1_ch_c2_orth + trust1_par_c2_orth

age_ch ~~ Adh1_val_par + QoLT1_ch

QoLT1_ch ~~ asthmaproblem_par

trust1_par_c ~~ asthmaproblem_par

'

## 15) Model estimation

fit_both <- sem(

model = model_both,

data = apim_data,

missing = "fiml", # handles missing data using Full Information Maximum Likelihood

estimator = "MLR" # robust to non-normality

)

fit_both <- sem(model_both, data = apim_data, missing = "fiml", estimator = "MLR")

**6)Comparisons between models**

# 17 (a) Linear vs. Full → test the added value of QUADRATIC terms

lavTestLRT(fit_apim, fit_both)

#18 (b) Quadratic-only vs. Full → test the added value of LINEAR terms

lavTestLRT(fit_apim2, fit_both)

## 19 Joint Wald test in the full model: are the quadratic effects zero?

lavTestWald(fit_both, constraints = "qa1 == 0; qp1 == 0")

## 20 Joint Wald test in the full model: are the linear effects zero?

lavTestWald(fit_both, constraints = "a1 == 0; p1 == 0")
